# Validation of the Spanish-language Cardiff Anomalous Perception Scale

**DOI:** 10.1371/journal.pone.0213425

**Published:** 2019-03-06

**Authors:** William Tamayo-Agudelo, María J. Jaén-Moreno, María O. León-Campos, Jorge Holguín-Lew, Rogelio Luque-Luque, Vaughan Bell

**Affiliations:** 1 Universidad Cooperativa de Colombia, Medellín, Colombia; 2 Division of Psychiatry, University College London, United Kingdom; 3 UGC Salud Mental, Hospital Universitario Reina Sofía, Córdoba, Spain; 4 Universidad de Antioquia, Medellín, Colombia; University of La Rioja, SPAIN

## Abstract

The Cardiff Anomalous Perceptions Scale (CAPS) is a psychometric measure of hallucinatory experience. It has been widely used in English and used in initial studies in Spanish but a full validation study has not yet been published. We report a validation study of the Spanish-language CAPS, conducted in both Spain and Colombia to cover both European and Latin American Spanish. The Spanish-language version of the CAPS was produced through back translation with slight modifications made for local dialects. In Spain, 329 non-clinical participants completed the CAPS along with 40 patients with psychosis. In Colombia, 190 non-clinical participants completed the CAPS along with 21 patients with psychosis. Participants completed other psychometric scales measuring psychosis-like experience to additionally test convergent and divergent validity. The Spanish-language CAPS was found to have good internal reliability. Test-retest reliability was slightly below the cut-off, although could only be tested in the Spanish non-clinical sample. The scale showed solid construct validity and a principal components analysis broadly replicated previously reported three component factor structures for the CAPS.

## Introduction

The Cardiff Anomalous Perceptions Scale (CAPS) is a self-report psychometric measure of hallucinatory experience that was first validated in 2006 [1] and was revalidated in a subsequent replication study [2] and has been widely used since. Since its first publication it has been used to examine the role of perceptual anomalies in paranoia [3], autism [4], psychosis in twins [5], the rubber hand illusion [6], hypomanic personality traits [7] and trauma [8], to name but a few areas of application.

Perhaps the most widely investigated aspect of hallucinations is where they appear as a component of psychosis [9]. Indeed, hallucinations can appear across the extended psychosis phenotype and can range from benign perceptual alterations to intense and distressing hallucinations with the latter more likely to be present in diagnosable psychiatric disorders [10]. The CAPS was designed to measure a range of hallucinatory experiences not limited to those found in the psychosis-spectrum, including, for example, alterations to sensory intensity and hallucinations from the neurological literature [1]. Nevertheless, it has been shown to be sensitive to psychosis-spectrum hallucinations can distinguish unselected patients with psychosis from the general population [1–2, 11] and psychotic patients with hallucinations from those without [12].

The CAPS has been translated and validated in Taiwanese [13], an initial validation study has been conducted with a Spanish-language version using non-clinical participants in Spain [14] and a factor analytic study using the Spanish-language scale has been conducted in non-clinical participants in Colombia [15]. However, a full validation study of a Spanish language version with both clinical and non-clinical participants has not yet been published.

This study reports a back translation and validation study of the Spanish-language Cardiff Anomalous Perceptions Scale in both non-clinical participants and patients with psychosis, conducted in both Spain and Colombia to cover both European Spanish and Latin American Spanish.

## Methods

All data, analysis scripts and copies of the questionnaires are freely available online at the following resource: https://osf.io/ekwgb/

### Translation and back translation

The CAPS was translated using the back translation method. The original English-language version was translated into standard Spanish by an independent professional translator following the conventions of the Royal Academy of the Spanish language (“Real Academia Española”). Feedback on potential uncertainties in translation and translation decisions was provided to the study authors. Subsequently, this initial Spanish-language version was translated back into English by two bilingual mental health professionals and the original and back-translated English versions compared. The Spanish and back-translated English versions were reviewed by an expert committee of the study authors. Due to differences in appropriate word choice between European Spanish and Latin American Spanish, a decision was made to produce two Spanish-language versions, based on local word preferences and different emphasis in meaning. For example, item 24 in English reads “Do you ever have the feeling of being uplifted, as if driving or rolling over a road while sitting quietly?” The verb ‘to drive’ differs between Spain (“conducir”) and Latin America (“manejar”) and so was changed for the local versions. The overall changes were minor and the two versions were then compared and judged to be equivalent in meaning. The scale was assessed by the research team in each country for equivalent face and content validity in comparison to the original version in English. Pre-testing was conducted through informally distributing the questionnaire to respondents naïve to the purpose of the study and receiving verbal feedback. No changes were made as a result of this phase.

### Procedure

The study was cross-sectional in design and data was collected by different researchers in Spain and Colombia. The relevant projects were reviewed and approved by ethics committees in each country: in Spain, the Research Ethics Committee of El Hospital Universitario Reina Sofía, in Córdoba, and in Colombia, the Bioethics Committee of La Universidad Cooperativa de Colombia–Medellín campus. la Universidad Cooperativa de Colombia Written informed consent was obtained from all participants before participation.

#### Participants

Participants were recruited in four groups. Nonclinical and clinical participants in Spain, and nonclinical and clinical participants in Colombia. All data was collected in 2011–2012.

Spain–Nonclinical sample: 324 individuals in Spain (Córdoba) were invited to participate. The participants were second-year medical students who had not yet received any teaching related to psychiatry or mental health. Participants were asked not to participate if they had any history of brain injury or psychotic illness. Participants who didn’t complete the full series of questionnaires were excluded, leaving 319 participants in the final analysis. 99 were male and 220 were female (mean age 20.1; SD = 2.4; range 18–38). Relationship status was reported as single (N = 262), married (N = 2), divorced / separated (N = 1), and with partner (N = 54). In line with the source of recruitment, all participants reported a university level education. Participants were additionally asked to indicate if they had a personal history of mental health issues (48 responded yes) and if they had a family history of mental health issues (55 responded yes). Following the original study,[1] after 6 months, participants were invited to complete the subset of questionnaires again of which data for 71 participants was collected. An initial analysis of data from this sample was originally reported in Jaén Moreno et al. [14]

Spain–Clinical sample: 40 patients with a clinical DSM-IV-TR diagnosis of schizophrenia participated in the study from an acute inpatient ward and outpatient clinic in Spain (Córdoba). Patients were not referred to the study if they were considered by the clinical team to have cognitive impairment that might interfere with informed consent. The sample consisted of 30 males and 10 females (mean age 32.5; SD = 5.60; range 21–39). Relationship status was reported as single (N = 31), married (N = 5), divorced / separated (N = 1), and with partner (N = 3). Education level was reported as primary education (N = 20), vocational training (N = 5), university (N = 6), completed undergraduate degree (N = 8) and postgraduate level (N = 1). All patients were taking antipsychotic medication at the time of participation and were assessed using the Brief Psychiatric Rating Scale as part of their clinical admission using the 0 to 6 scaling system [16] resulting in a mean score of 16.95 (SD = 8.05).

Colombia–Nonclinical sample: 209 individuals from the general population of Colombia (Medellín) participated in the study and were invited by distributing invitations to domestic residences and offices in the city. After removal of incomplete data, 190 participants, were retained for the final analysis. Participants were asked not to participate if they had a history of psychiatric illness or traumatic brain injury. The sample includes 79 males and 111 females (mean age 36.02; SD = 15.39; range 17–99). Relationship status was reported as single (N = 98), married (N = 59), divorced / separated (N = 9), and with partner (N = 18), widower (N = 5), and didn’t respond (N = 1). Education was not recorded for this sample. An initial analysis of data from this sample was originally reported in Tamayo et al. [15]

Colombia–Clinical sample: A total of 21 patients, from an outpatient clinic for patients diagnosed with schizophrenia in Colombia (Medellín) participated in the study. The patients were diagnosed by the clinical team using DSM-IV criteria. The sample includes 13 males and 8 females (mean age 40.43; SD = 11.31; range 22–63). Relationship status was reported as single (N = 16), married (N = 4) and divorced / separated (N = 1). Education was not recorded for this sample. All patients were taking antipsychotic medication at the time of participation.

#### Measures

All measures were presented in their Spanish-language version. Not all measures were presented to all samples and where measures were not completed by all participants, the specific groups to which they were presented are described below.

Cardiff Anomalous Perceptions Scale (CAPS): a 32-item self-report scale designed to measure perceptual anomalies and hallucinatory experience that has already been validated in English in clinical and nonclinical populations [1–2]. Each of the 32 items involves a question related to a specific hallucinatory experience to which the participant can answer ‘yes’ or ‘no’. If the participant answers ‘yes’ they are asked to rate how distressing, how intrusive and how often the experience occurs on separate 1–5 rated Likert scales. The scale total is calculated as the total number items responded to with ‘yes’ (possible range 0–32) and the subscale totals are calculated as the total of the subscale items (possible range 0–160). The Spanish language versions of the CAPS used in this study have been made freely available at the following link: https://osf.io/ekwgb/

Revised Launay-Slade Hallucination Scale (RLSHS). A 12-item self-report scale that measures the predisposition to hallucinatory experiences [17]. This was used in all samples except the clinical sample from Spain. The validated Spanish-language version of the RLSHS was used in this study [18–19].

Brief Oxford-Liverpool Inventory of Feelings and Experiences (O-LIFE-R): a self-report scale designed to measure schizotypy [20]. The brief version has 40 items that require a ‘yes / no’ response. It has four subscales with measure unusual experiences, cognitive disorganisation, introverted anhedonia and impulsive nonconformity. Total and subscale scores and calculated by summing the relevant affirmative answers. This was only used in the non-clinical sample from Spain. The validated Spanish-language translation of the scale was used in this study [21–22].

Peters et al Delusions Inventory, 21 item version (PDI-21): a self-report scale designed to measure delusional ideation and magical thinking [23]. It is scored in a similar way to the CAPS with 21 questions that requires a ‘yes / no’ and three subscales that measure preoccupation, conviction and distress which participants are asked to complete for each item that they respond to with a ‘yes’ response. This was only used in the non-clinical sample from Spain. The validated Spanish-language version of this scale was used in this study [24–25].

#### Analysis

All statistical analysis was completed using SPSS 25 and R version 3.4.4. Internal reliability was calculated using Cronbach’s alpha and Omega [26]. Test-retest reliability was calculated using a Pearson correlation in the non-clinical sample for Spain who completed the scale twice. Discriminant validity was tested using an independent samples t-test by comparing clinical and non-clinical groups from both countries. Convergent validity was tested by comparing CAPS and RLSHS total score using a Pearson correlation coefficient in non-clinical groups in both countries.

Due to the additional measures in the sample from Spain, convergent validity was additionally tested in this non-clinical sample by comparing CAPS total and PDI-21 total using a Pearson correlation. Convergent and divergent validity in the Spanish non-clinical sample by comparing CAPS total and O-LIFE subscale scores using Pearson correlations with the prediction than the CAPS would selectively correlate with the O-LIFE unusual experiences subscale but only weakly or non-significantly with the other schizotypy subscales.

We also completed a 2x2 between-subjects ANOVA comparing CAPS Total score with two factors, each with two levels: country (Spain vs Colombia) and clinical groups (clinical vs non-clinical) to investigate whether there was an interaction between country and clinical group.

Finally, we conducted a principal components analysis extracting three factors following the methods from the original validation study published in Bell et al. [1], using the CAPS main items with direct oblimin rotation.

## Results

### Descriptive statistics

Means and standard deviations for scale scores are reported in Table 1. Kurtosis and skewness for all variables are reported in the supplementary material in S1 Table. As expected, clinical groups scored higher than non-clinical groups on the total score and all subscales of the CAPS in both the samples from Spain and Colombia.

**Table 1:** Scale means and standard deviations for nonclinical and clinical samples in Spain and Colombia.

		*CAPS*					*O-LIFE*					
*Group*	*N*	*Total*	*Distress*	*Intrusiveness*	*Frequency*	*RLSHS*	*Total*	*UE*	*CD*	*IA*	*IN*	*PDI-21*
*Spain Nonclinical*	319	8.79 (5.44)	17.33 (14.67)	15.35 (12.65)	17.66 (12.44)	18.50 (4.46)	11.85 (4.72)	1.94 (1.67)	5.28 (2.58)	1.32 (1.72)	3.24 (1.85)	4.24 (2.63)
*Spain Psychosis*	40	11.73 (6.06)	34.25 (21.09)	32.40 (20.58)	30.60 (20.75)	-	-	-	-	-	-	-
*Colombia Nonclinical*	190	7.28 (5.74)	18.16 (16.79)	16.77 (15.97)	17.76 (15.32)	23.10 (6.85)	-	-	-	-	-	-
*Colombia Psychosis*	21	15.05 (7.53)	50.67 (36.55)	45.90 (36.55)	47.05 (31.45)	27.95 (7.77)	-	-	-	-	-	-
*Combined Nonclinical*	509	8.23 (5.59)	17.64 (15.48)	15.88 (13.98)	17.70 (13.57)	20.22 (5.90)	11.85 (4.72)	1.94 (1.67)	5.28 (2.58)	1.32 (1.72)	3.24 (1.85)	4.23 (2.64)
*Combined* *Psychosis*	61	12.87 (6.73)	39.90 (28.22)	37.05 (27.61)	36.26 (25.92)	27.95 (7.77)	-	-	-	-	-	-

CAPS = Cardiff Anomalous Perceptions Scale. RLSHS = Revised Launay Slade Hallucinations Scale. O-LIFE = Oxford-Liverpool Inventory of Life and Experiences. PDI-21 = Peters et al Delusions Inventory. UE = Unusual experiences subscale. CD = Cognitive disorganisation subscale. IA = Introvertive anhedonia subscale. IN = Impulsive nonconformity subscale.

### Reliability

In the sample collected from Spain, the Cronbach’s alpha for the non-clinical sample was 0.834 and the coefficient Omega was 0.837 (95% CI 0.81–0.86). In the data from Colombia, the Cronbach’s alpha for the non-clinical sample was 0.869 and the coefficient Omega was 0.874 (95% CI 0.84–0.90). For the combined non-clinical sample from both countries the Cronbach’s alpha was 0.848 and the coefficient Omega was 0.851 (95% CI 0.83–0.87). Overall, these analyses indicate good evidence for internal reliability. Test-retest reliability examined using Pearson correlation on 71 participants for the non-clinical sample from Spain over a six month period was 0.61. A typical cut-off for test-retest reliability is 0.7 [27] and indicates a test-retest reliability marginally below the cut-off.

### Criterion-related evidence of validity

CAPS total score and subscale scores between clinical and non-clinical groups were compared using two-tailed independent samples t-tests and all showed a significant difference in that patients with psychosis scored significantly more on the CAPS than non-clinical groups.

All CAPS scores were significantly different between non-clinical participants and patients with psychosis in the sample from Spain: CAPS total (t = 3.181, p = 0.002, d = 0.53), CAPS distress (t = 6.507, p < 0.0001, d = 1.09), CAPS Intrusiveness (t = 7.399, p < 0.0001, d = 1.24), CAPS frequency (t = 5.673, p < 0.0001, d = 0.95).

All CAPS scores were significantly different in the sample from Colombia: CAPS total (t = 5.685, p < 0.0001, d = 1.32), CAPS distress (t = 7.225, p < 0.0001, d = 1.67), CAPS Intrusiveness (t = 6.691, p < 0.0001, d = 1.55), CAPS frequency (t = 7.271, p < 0.0001, d = 1.68).

Similarly, all CAPS scores were significantly different in the combined sample: CAPS total (t = 5.986, p < 0.0001, d = 0.81), CAPS distress (t = 9.509, p < 0.0001, d = 1.29), CAPS Intrusiveness (t = 9.777, p < 0.0001, d = 1.33), CAPS frequency (t = 8.925, p < 0.0001, d = 1.21).

These results demonstrate that CAPS scores distinguish between non-clinical groups and patients with psychosis, indicating good discriminant validity.

### Evidence of validity based on relationships with measures of other variables

Using non-clinical samples, CAPS total score significantly correlated with RLSHS score in both the sample from Spain (r = 0.561, p < 0.0001) and the sample from Colombia (r = 0.406, p < 0.0001) and the total sample (r = 0.443, p < 0.0001).

Additionally, data from the Brief O-LIFE scale was collected from the non-clinical sample from Spain. CAPS total score correlated moderately with the O-LIFE unusual experiences subscale (r = 0.5, p < 0.0001), weakly with the cognitive disorganisation subscale (r = 0.254, p < 0.0001), weakly with the impulsive non-conformity scale (r = 0.216, p < 0.0001) and weakly and non-significantly with the introvertive anhedonia scale (r = 0.088, p = 0.115). PDI-21 data was also collected in the non-clinical sample Spain and correlated significantly with the CAPS total score (r = 0.569, p < 0.0001).

These results indicate that the CAPS shows convergent validity with scales that measure similar hallucinatory, psychosis-related constructs and divergent validity from scales that measure dissimilar constructs.

### Interaction between countries and clinical groups

A two-way 2x2 between-subjects ANOVA was conducted on the CAPS main score. The first factor was country (Spain vs Colombia), the second factor was clinical group (non-clinical vs clinical). There was no main effect for country (F_(1,566)_ = 1.272, p = 0.260, partial eta squared = 0.002) indicating that overall CAPS scores were not significantly different between Spain and Colombia. There was a main effect of clinical group (F_(1,566)_ = 43.973, p < 0.0001, partial eta squared = 0.072) in that participants with psychosis scored significantly higher than participants in the non-clinical groups. There was a significant interaction (F_(1,566)_ = 8.940, p = 0.003, partial eta squared = 0.016) in that patients from Colombia scored above patients from Spain, but members of the non-clinical population in Colombia scored below the non-clinical population in Spain. When tested with post-hoc t-tests, the difference between non-clinical populations in Spain and Colombia was significant (t = 2.953, p = 0.003) but the difference between clinical populations was not (t = 1.870, p = 0.066). However, we note the difference between non-clinical participants on CAPS main score although significant was small in magnitude (a mean difference of 1.51 points).

### Evidence of validity based on internal structure

Non-clinical samples from Spain and Colombia were combined for a principal components analysis (PCA), meaning data from 509 non-clinical participants were entered into this analysis. The Kaiser-Meyer-Olkin value was 0.856 and Bartlett’s Test of Sphericity reached statistical significance, supporting the suitability of the data for this analysis. We completed a PCA using direct oblimin rotation, requesting three factors based on the factor structure found in the original validation study [1]. The three factors explained 29.33% of the total variance. The factor loadings for items in the scale are displayed in Table 2.

**Table 2:** CAPS items and factor loading after principal components analysis (oblimin rotation) of nonclinical sample responses.

		Component		
Item	Item text	1	2	3
15	Do you ever find that sensations happen all at once and flood you with information?	0.60		
22	Do you ever look in the mirror and think that your face seems different from usual?	0.56		
5	Do you ever experience unusual burning sensations or other strange feelings in or on your body?	0.52		
3	Do you ever hear your own thoughts repeated or echoed?	0.52		
26	Do you ever think that everyday things look abnormal to you?	0.48		
17	Do you ever have difficulty distinguishing one sensation from another?	0.46		
23	Do you ever have days where lights or colours seem brighter or more intense than usual?	0.43		
10	Do you ever have the sensation that your limbs might not be your own or might not be properly connected to your body?	0.41		
4	Do you ever see shapes, lights or colours even though there is nothing really there?	0.40		
9	Do you ever have the sensation that your body, or a part of it, is changing or has changed shape?	0.38		
1	Do you ever notice that sounds are much louder than they normally would be?	0.35		
24	Do you ever have the feeling that of being uplifted, as if driving or rolling over a road while sitting quietly?	0.31		
28	Have you ever heard two or more unexplained voices talking with each other?		0.68	
13	Do you ever hear voices saying words or sentences when there is no-one around that might account for it?		0.62	
31	Do you ever see things that other people cannot?		0.60	
11	Do you ever hear voices commenting on what you are thinking or doing?		0.57	
2	Do you ever sense the presence of another being, despite being unable to see any evidence?		0.50	
32	Do you ever hear sounds or music that people near you don’t hear?		0.39	
6	Do you ever hear noises or sounds when there is nothing about to explain them?	0.31	0.33	
12	Do you ever feel that someone is touching you, but when you look nobody is there?		0.31	
7	Do you ever hear your own thoughts spoken aloud in your head, so that someone near might be able to hear them?			
21	Do you ever think that food or drink tastes much stronger than it normally would?			-0.73
18	Do you ever smell everyday odours and think that they are unusually strong?			-0.68
25	Do you ever find that common smells sometimes seem unusually different?			-0.62
30	Do you ever notice that food or drink seems to have an unusual taste?			-0.62
8	Do you ever detect smells which don’t seem to come from your surroundings?			-0.51
29	Do you ever notice smells or odours that people next to you seem unaware of?			-0.49
20	Do you ever find that your skin is more sensitive to touch, heat or cold than usual?			-0.41
14	Do you ever experience unexplained tastes in your mouth?			-0.39
19	Do you ever find the appearance of things or people seems to change in a puzzling way, e.g. distorted shapes or sizes or colour?			-0.38
16	Do you ever find that sounds are distorted in strange or unusual ways?			
27	Do you ever find that your experience of time changes dramatically?			

Factor loadings below 0.3 are not shown

The factor structure broadly replicated the original factor structure reported in Bell et al. [1]. Two factors could be clearly interpreted as “clinical psychosis” and “chemosensation” and despite one component showing a significant overlap of items with the Bell el al. [1] “temporal lobe experience” scale, we were equivocal whether this was the best interpretation and this may be better interpreted as “non-clinical hallucinatory experience” factor.

## Discussion

We report a validation study of the Spanish-language version of the Cardiff Anomalous Perception scale in both Colombia and Spain, finding that this translated version of the scale is both reliable and valid. The CAPS showed good internal reliability, discriminant validity, convergent and divergent validity, and broadly reproduced the same three component factor structure as the original validation study. The test-retest reliability was marginally below the level of acceptability but it was only possible to test it in the non-clinical population in one country.

One notable finding was an interaction between country and participant group, finding that patients with psychosis from Colombia typically scored higher on the CAPS than patients with psychosis from Spain, and the non-clinical population in Colombia typically scored lower than the non-clinical population from Spain. However, when the CAPS main score for clinical groups was tested with a direct post-hoc comparison, the difference between the patient sample from Colombia and patient sample from Spain was not significant and the difference between non-clinical groups, although significant, was of small effect. Given these results, we suggest it is unlikely that this reflects a true population difference, although we note both age differences (the non-clinical sample from Spain was markedly younger than the non-clinical population from Colombia) and cultural differences may play a part in differences in hallucinatory experience, and need to be investigated further to fully confirm the equivalence of populations in this regard. However, the extent to which the CAPS measures similar constructs across populations is best tackled using a measurement invariance analyses [28] and future research should test this as an additional aspect of the scale’s validity across countries and cultures.

The principal components analysis broadly replicated the three-component solution first reported in the original validation study [1]. In this original study, the three factors were labelled “clinical psychosis”–reflecting experiences most associated with clinically diagnosable psychosis spectrum disorders, “temporal lobe experience”–reflecting hallucinatory experiences commonly associated with disturbances to the temporal lobe, such as temporal lobe epilepsy, and “chemosensation”–which reflected alterations to the senses of taste and smell.

In this study, the “chemosensation” factor was clearly replicated, as was a factor that seemed to reflect experiences more common in psychosis. Although the remaining factor overlapped considerably with the original “temporal lobe experience” factor some of the characteristic ‘temporal lobe’ experiences were missing (e.g. items 24, 27). In retrospect, it may be that this factor better represents ‘non-clinical hallucinatory experiences’ factor rather than temporal lobe-related experiences *per se*. Notably, this original three factor solution was not replicated a subsequent validation study of the CAPS [2] or in a study of non-clinical participants in Colombia [15]. One marked difference between the studies that have found a three factor solution and those that haven’t, is sample size–with positive studies including more than 300 participants, and negative studies including approximately 200 or less. One hypothesis is that the factor structure of anomalous experiences measured by the CAPS is unstable and varies between populations, another is that it is present but not detectable except with larger sample sizes, suggesting it may be present but only weakly so.

It is also worth noting some limitations to this study. Although this study was conducted in line with recommendations for cross-cultural and cross-language adaptation of self-report measures [29–30], the most complete process has been described by Beaton et al. [31] and involves two translators–one familiar with the construct being measured by the scale and one naïve to its psychometric purpose–to give the best chance of highlighting potential challenges or subtleties in translation. In our study we used only one construct naïve translator for the initial translation that may have under-detected challenges in translation particularly where the language intended to capture specific clinical phenomena, although the fact that separate expert committees in Spain and Colombia checked and commented on the resulting translation with respect to country specific language may have mitigated this to some extent. We also note that the non-clinical sample in Spain consisted of medical students who may be non-typical in several respects in comparison to other members of the population–in terms of education, cognitive ability, but perhaps most relevant to this study, high levels of stigma towards mental health problems [32] potentially affecting how they responded to the items on the scales. Finally, we tested test-retest reliability over a six-month time period based on the design of the original validation study [1]. However, this retest period was arbitrarily selected in the original study and it is possible that true variation in the presence and intensity of hallucinations in the general population, as has been found in experience sampling studies of psychosis,[33] may mean that a shorter time period is necessary for adequate test-retest reliability assessment.

Nevertheless, the data presented here provide good evidence that the Spanish-language version of the CAPS is reliable and valid in both Spain and Colombia, when tested with a sample that includes nonclinical participants and patients with psychosis.

## Supporting information

S1 Table SupplementaryInformation. (DOCX)
