# Supplementary material for: Validation of the Spanish-language Cardiff Anomalous Perception Scale
Source: PLoS One. 2019 Mar 6;14(3):e0213425. doi: 10.1371/journal.pone.0213425 (PMC6402668; doi:10.1371/journal.pone.0213425)
Supplement: S1 Table — (DOCX) [file pone.0213425.s001.docx]

Supplementary information for

Validation of the Spanish-language Cardiff Anomalous Perception Scale

William Tamayo-Agudelo^1,2^, María J. Jaén-Moreno^3^, María O. León-Campos^2^, Jorge Holguín-Lew^4^, Rogelio Luque-Luque^3^, Vaughan Bell^2*^

| Country | | | *Age* | *CAPS Total* | *CAPS Distress* | *CAPS Intrus* | *CAPS Freq* | RLSHS | *OLIFE Total* | *OLIFE EU* | *OLIFE CD* | *OLIFE IA* | *OLIFE IN* | *PDI-21 Total* | *PDI Distress* | *PDI Preocc* | *PDI-21 Believe* | *BPRS Total* |
| --- | --- | --- | --- | --- | --- | --- | --- | --- | --- | --- | --- | --- | --- | --- | --- | --- | --- | --- |
| Spain | General Pop | Kurtosis | 38.568 | 0.061 | 3.918 | 2.924 | 2.130 | 0.781 | 0.782 | 1.133 | -0.810 | 2.750 | -0.284 | 0.961 | 5.515 | 6.609 | 2.835 |  |
|  |  | Skewness | 5.286 | 0.456 | 1.642 | 1.445 | 1.054 | 0.878 | 0.482 | 1.095 | -0.088 | 1.602 | 0.308 | 0.778 | 1.743 | 1.893 | 1.186 |  |
|  | Psychotic | Kurtosis | -0.777 | -0.250 | 0.518 | 0.543 | 2.286 |  |  |  |  |  |  |  |  |  |  | -0.843 |
|  |  | Skewness | -0.727 | 0.630 | 1.011 | 1.063 | 1.423 |  |  |  |  |  |  |  |  |  |  | 0.017 |
|  | Total | Kurtosis | 5.391 | 0.107 | 3.344 | 3.603 | 4.387 | 0.781 | 0.782 | 1.133 | -0.810 | 2.750 | -0.284 | 0.961 | 5.515 | 6.609 | 2.835 | -0.843 |
|  |  | Skewness | 2.509 | 0.502 | 1.613 | 1.637 | 1.516 | 0.878 | 0.482 | 1.095 | -0.088 | 1.602 | 0.308 | 0.778 | 1.743 | 1.893 | 1.186 | 0.017 |
| Colombia | General Pop | Kurtosis | 2.387 | -0.159 | 2.160 | 1.885 | 0.016 | -0.628 |  |  |  |  |  |  |  |  |  |  |
|  |  | Skewness | 1.362 | 0.692 | 1.331 | 1.353 | 0.865 | 0.313 |  |  |  |  |  |  |  |  |  |  |
|  | Psychotic | Kurtosis | -0.707 | -0.307 | 1.195 | 1.895 | 3.603 | -0.395 |  |  |  |  |  |  |  |  |  |  |
|  |  | Skewness | 0.317 | 0.036 | 1.013 | 1.268 | 1.461 | 0.475 |  |  |  |  |  |  |  |  |  |  |
|  | Total | Kurtosis | 2.211 | 0.137 | 6.593 | 8.179 | 7.295 | -0.434 |  |  |  |  |  |  |  |  |  |  |
|  |  | Skewness | 1.278 | 0.769 | 2.047 | 2.244 | 1.902 | 0.374 |  |  |  |  |  |  |  |  |  |  |
| Total | General Pop | Kurtosis | 7.636 | -0.115 | 3.106 | 2.627 | 1.063 | 0.364 | 0.782 | 1.133 | -0.810 | 2.750 | -0.284 | 0.961 | 5.515 | 6.609 | 2.835 |  |
|  |  | Skewness | 2.532 | 0.516 | 1.510 | 1.448 | 0.969 | 0.869 | 0.482 | 1.095 | -0.088 | 1.602 | 0.308 | 0.778 | 1.743 | 1.893 | 1.186 |  |
|  | Psychotic | Kurtosis | 1.357 | -0.370 | 2.713 | 3.614 | 4.172 | -0.395 |  |  |  |  |  |  |  |  |  | -0.843 |
|  |  | Skewness | 0.897 | 0.460 | 1.407 | 1.599 | 1.651 | 0.475 |  |  |  |  |  |  |  |  |  | 0.017 |
|  | Total | Kurtosis | 6.156 | 0.073 | 6.279 | 8.204 | 7.424 | 0.569 | 0.782 | 1.133 | -0.810 | 2.750 | -0.284 | 0.961 | 5.515 | 6.609 | 2.835 | -0.843 |
|  |  | Skewness | 2.217 | 0.587 | 1.956 | 2.155 | 1.852 | 0.920 | 0.482 | 1.095 | -0.088 | 1.602 | 0.308 | 0.778 | 1.743 | 1.893 | 1.186 | 0.017 |

**Table S1. Kurtsosis and Skewness for Study Variables**

CAPS = Cardiff Anomalous Perceptions Scale. RLSHS = Revised Launay Slade Hallucinations Scale. OLIFE = Oxford-Liverpool Inventory of Life and Experiences. UE = Unusual experiences subscale. CD = Cognitive disorganisation subscale. IA = Introvertive anhedonia subscale. IN = Impulsive nonconformity subscale. PDI-21 = Peters et al Delusions Inventory.
